# *Eucommia ulmoides* Leaf Extract Ameliorates Steatosis Induced by High-fat Diet in Rats by Increasing Lysosomal Function

**DOI:** 10.3390/nu11020426

**Published:** 2019-02-18

**Authors:** Geum-Hwa Lee, Hwa-Young Lee, Sun-Ah Park, Tai-Sun Shin, Han-Jung Chae

**Affiliations:** 1 Non-Clinical Evaluation Center, Biomedical Research Institute, Chonbuk National University Hospital, Jeonju, Chonbuk 54907, Korea; heloin@jbnu.ac.kr (G.-H.L.); topaz6183@jbnu.ac.kr (S.-A.P.); 2 Department of Pharmacology and New Drug Development Institute, Chonbuk National University Medical School, Jeonju, Chonbuk 54896, Korea; youngat84@gmail.com; 3 Division of Food and Nutrition, College of Human Ecology, Chonnam National University, Gwang-ju 61186, Korea; shints@chonnam.ac.kr

**Keywords:** NAFLD, ER stress, *Eucommia ulmoides* leaf, autophagy, mTOR

## Abstract

The recent discovery that the impairment of autophagic flux in non-alcoholic fatty liver disease (NAFLD) might be a strong determining factor in steatosis suggests the potential of therapeutic control of autophagic flux with natural agents in restoring NAFLD. We investigated the potential of *Eucommia ulmoides* leaf extract (EUL) to control dyslipidemia in NAFLD. EUL supplementation (200 mg/kg) promoted recovery from high fat diet (HFD)-induced lipid dysmetabolism. This hepatoprotective efficacy was accompanied by suppression of endoplasmic reticulum (ER) stress, enhancing lysosomal functions, and thereby increasing autophagic flux. We found a strong indication that inhibition of the mTOR-ER stress pathway was related to the enhanced autophagic flux. However, the direct antioxidative effect of EUL on cytoprotection cannot be ruled out as a significant contributing factor in NAFLD. Our findings will aid in further elucidating the mechanism of the anti-steatosis activity of EUL and highlight the therapeutic potential of EUL in the treatment of NAFLD.

## 1. Introduction

Nonalcoholic fatty liver disease (NAFLD) is a spectrum of liver diseases ranging from non-alcoholic steatohepatitis, liver fibrosis, cirrhosis, and liver cancer to steatosis. NAFLD is characterized by excessive lipid accumulation in hepatocytes [1,2]. It is currently the most common chronic liver disease worldwide. NAFLD is one of the major pathological processes that occur in the early stages of liver diseases, such as liver fibrosis and inflammation [2,3]. Recent evidence implicates the disruption of endoplasmic reticulum (ER) homeostasis, or ER stress, in the development of hepatic steatosis [4,5]. ER stress may lead to the activation of various intracellular stress pathways that can initiate or exacerbate insulin resistance (IR) and inflammation and, in some cases, culminate in hepatocyte death and liver damage, all of which are important in the pathogenesis of NAFLD [6]. Therefore, there is an urgent need to develop therapeutic/preventive agents against NAFLD that regulate ER stress. Furthermore, a specific strategy against ER stress, i.e., the enhancement of lysosomal activity leading to protein degradation and ultimately, lessening the protein-folding load, needs to be established in hepatic dyslipidemia. Leaves of *Eucommia ulmoides* Oliver (EUO) have become a popular functional health food and plant medicine material in China and Japan. Du-zhong tea, the aqueous extract of EUO leaves (EUL), is known as a functional health food and is commonly used in the treatment of hypertension, hypercholesterolemia, and fatty liver. We have reported that lysosomal activation induced by cortex extract of EUO in NAFLD possibly contributed to the recovery to hepatic normal status through ER stress regulation in hepatic dyslipidemia [7]. Lysosomal protein degradation is considered a physiologic and adaptive process, also termed autophagy. ER stress, cellular organelle degradation, and autophagy have been frequently studied in hepatic dysmetabolism. In addition, high-nutrient-based ER stress is expected to be linked to protein hyperfolding and excessive ER load, leading to ER oxidative folding, redox imbalance, and ROS accumulation. Based on recently reported characteristics, EUO is expected to control ER stress through lysosomal activation, lessening the protein-folding load. Given that little is known about how the natural product EUL impacts ER stress in the liver, studying the effects of EUL on the autophagic mechanism in hepatic lipid metabolism and hepatic ER stress would be of interest. In this study, we aimed to investigate the effect of EUL supplementation on high-fat diet-induced NAFLD in rats and to explore whether it contributed to hepatoprotection through regulation of the lysosomal-autophagy pathway and/or by decreasing ER stress.

## 2. Materials and Methods

### 2.1. Extraction and Purification of EUL

The leaves of *Eucommia ulmoides* were collected from Yeongcheon, Gyeongbuk, Korea, and authenticated by Dr. Tai-Sun Shin, Division of Food and Nutrition, College of Human Ecology, Chonnam National University, Gwang-ju, Korea. A voucher specimen (ID201801) has been deposited at the Herbarium of Department of Pharmacology, Chonbuk National University Medical School, Jeonju, Korea. The leaves were air-dried and powdered. The powder (1000 g) was extracted with 5000 mL of distilled water for 2 h at 121 °C. The EUL was centrifuged at 5000 × *g* for 20 min at 4 °C (Himac CR-22F; Hitachi Koki Co., Ltd., Tokyo, Japan) and the supernatant was filtered through Whatman No. 1 filter paper (Sigma-Aldrich, St. Louis, MO, USA). The filtrate was concentrated in a rotary evaporator and lyophilized in a freeze dryer (Ilshin Lab Co., Ltd., Seoul, Korea) [8]. The polyphenols of EUL were extracted, and then analyzed for their chemical composition, as reported previously [8]. In our previous study, the composition analysis revealed that it contained aucubin (8.6 ± 0.06 mg/g), geniposidic acid (55.2 ± 0.37 mg/g), and chlorogenic acid (11.63 ± 0.15 mg/g) [8].

### 2.2. Animals and Experimental Set-Up

Male Sprague-Dawley rats weighing 240–260 g were obtained from Orient Science Co (Seongnam, Korea). The rats were maintained on a 12 h : 12 h light:dark cycle (lights on at 06:00) in stainless-steel-wire-bottomed cages and were acclimated to laboratory conditions for at least 1 week before experiments. The rats were divided in the following treatment groups: a normal chow diet (NCD) group, which was fed a standard diet; an NCD plus EUL group, which received 200 mg/kg EUL; a 60% HFD group, which was fed a HFD; and a 60% HFD plus EUL group, which received EUL (100 mg/kg or 200 mg/kg). The NCD groups (*n* = 10 each) were fed a standard diet, whereas the HFD groups (*n* = 10 each) were fed a calorie-rich diet of 1% cholesterol, 18% lipid (lard), 40% sucrose, 1% AIN-93G vitamins, and 19% casein, with the same fiber and mineral contents as the standard diet. The experiment was terminated after 10 weeks. Rats were anesthetized with diethyl ether (Sigma, St. Louis, MO, USA) and cervical dislocation was performed. All animal procedures in this study were approved by the Institutional Animal Care and Use Committee of Chonbuk National University laboratory animal center (IACUC, CBNU 2017-0034), and all efforts were made to minimize animal suffering.

### 2.3. Biochemical Analyses

Plasma levels of total cholesterol, triglyceride (TG), aspartate aminotransferase (AST), alanine aminotransferase (ALT) (Asan Pharmaceutical, Seoul, Korea), and low-density lipoprotein (LDL) cholesterol (BioVision, Milpitas, CA, USA) were measured using commercially available kits. To quantify liver TG, liver tissues were homogenized and extracted in a mixture of chloroform, methanol, and distilled water (2:1:1, *v*/*v*/*v*). The TG concentration was measured with a TG Assay Kit (Asan Pharmaceutical, Seoul, Korea) and was expressed as milligrams of TG per 100 mg of liver tissue. Glutathione peroxidase (GPx), superoxide dismutase (SOD), and catalase (CAT) activities, and the malondialdehyde (MDA) level were measured using commercial assay kits (Cayman Inc., Ann Arbor, MI, USA) according to the manufacturer’s protocols.

### 2.4. Immunoblotting

Liver homogenates and cells were lysed in ice-cold RIPA buffer (50 mM Tris HCl (pH 7.4), 250 mM NaCl, 1% Nonidet P-40, and protease inhibitor cocktail). The protein samples (50 µg) were separated by 4%–12% sodium dodecyl sulfate polyacrylamide gel electrophoresis and then transferred onto PVDF membranes. After blocking with 5% BSA or 5% non-fat dry milk, the membranes were incubated overnight with the following primary antibodies: anti-p-mTOR, anti-mTOR, anti-p-p70s6kinase, anti-p70s6kinase, anti-p-4EBP-1, anti-4EBP-1, anti-CHOP, anti-p-eIF2α, and anti-eIF2α (Cell Signaling Technology, Boston, MA, USA), anti-ubiquitin, anti-GRP78, anti-p-PERK, anti-PERK, anti-Bax, anti-lamp-1, anti-Tom20, anti-cathepsin B, and β-actin (Santa Cruz Biotechnology, Santa Cruz, CA, USA), anti-p62 (SQSTM1; MBL International, Woburn, MA, USA), and anti-LC3II (Novus Biologicals, Littleton, CO, USA). Immunoreactive bands were visualized using the ECL Western blotting protocol (Bio-Rad). Finally, the membranes were exposed to imaging film (Kodak BioFlexEcono Scientific Supplies, Citrus Heights, CA, USA), after which the film was developed using a Kodak X-OMAT 1000A Processor. Densitometric analysis of the bands was conducted using the Image J software (NIH, Bethesda, MD, USA).

### 2.5. Lysosomal Fractionation

Lysosomal fractionation was conducted using the Lysosome Isolation Kit (Sigma, St. Louis, MO, USA, LYSISO1). Briefly, liver tissues and Hep G2 cells were harvested, washed, trypsinized, and pelleted. After another wash, the cells were suspended in extraction buffer and subjected to Dounce homogenization (Pestle B) followed by centrifugation (1000 × *g* for 10 min). The supernatant was collected and centrifuged at 20,000 × *g* for 20 min to pellet the crude lysosomal fraction. Both from the pellet and the supernatant, aliquots were collected for lysosomal enzyme activity and western blot analysis. The remaining crude lysosomal fraction was placed on an Optiprep step gradient and ultra-centrifuged at 150,000 × *g* for 4 h. Finally, both the pellet and supernatant (purest lysosomal fraction) were collected for analysis. Protease inhibitor cocktail was added to all samples.

### 2.6. Cathepsin B Activity

Cathepsin B (CtsB) activity was measured using the Abcam CtsB activity assay kit per the manufacturer’s instructions. Briefly, cathepsins (0.15–15 nM) were incubated with their corresponding substrates (Ac-RR-AFC for CtsB) at a final concentration of 20 μM in a pH 5 buffer. For experiments with CtsB, the buffer also contained 5 mM EDTA and 5 mM DTT. Reactions were carried out in 96-well flat-bottom microplates containing 200 μL of solution at 37 °C, and were read out using a microplate reader (Spectra Max M5, Sunnyvale, CA, USA). For CtsB, excitation and emission wavelengths were 400 and 505 nm, respectively.

### 2.7. OxyBlot Assay

Carbonylated proteins were detected using the OxyBlot^®^ Kit (Millipore, Bedford, MA, USA) according to the manufacturer’s protocol.

### 2.8. Cellular Reactive Oxygen Species (ROS) Detection

Intracellular levels of ROS, including O_2_^−^, were measured by means of the oxidation-sensitive fluorescent probe dye dihydroethidium (DHE; Ex/Em = 518/605 nm; Invitrogen/Molecular Probes). Liver tissue sections were washed in phosphate-buffered saline (PBS) and incubated with 20 μM DHE at 37 °C for 30 min. For quantitative determination, tissue sections were scanned under a microscope with a 20× objective. The cell area was quantified via DHE staining using Zeiss LSM 510 META confocal microscope (Carl Zeiss, Jena, Germany) with subtraction of background fluorescence.

### 2.9. Analysis of Lipid Peroxidation

Lipid peroxides were extracted from samples with chloroform and extraction buffer (BioVision, Milpitas, CA, USA). The mixture was left at room temperature for 5 min to react and develop color. The absorbance at 500 nm was read using a 96-well-plate spectrometer (SpectraMax 190, Sunnyvale, CA, USA). 13-Hydroperoxyoctadecadienoic acid was used as a standard. Cellular levels of lipid peroxide were calculated as described by the manufacturer.

### 2.10. Histological and Immunohistochemical Analyses

Liver tissue was fixed in 10% buffered formaldehyde and embedded in paraffin. For histological observation, 4-µm sections were deparaffinized in xylene and rehydrated in an alcohol gradient, and then stained with hematoxylin and eosin (H&E). For immunohistochemistry, the sections were dewaxed and rehydrated using the above method. After dewaxing, the sections were incubated overnight at 4°C with anti-4-HNE antibody (Millipore Corporation, Billerica, MA, USA). Then, the sections were washed three times with PBS, and endogenous peroxidase was blocked with 3% H_2_O_2_ in absolute methanol for 30 min at room temperature. Next, the sections were incubated with EnVision+ System-HRP (DAKO, Glostrup, Denmark) for 45 min at room temperature. Finally, the reaction products were stained with diaminobenzidine (DAB), counterstained with Mayer’s hematoxylin, and mounted with Eukitt mounting medium after drying. Images were acquired and analyzed with ImageJ.

### 2.11. Oil Red O Staining

Oil red O staining was performed as previously described [9]. Liver sections were washed with PBS twice, fixed in 4% formaldehyde in PBS for 30 min, and stained with 0.6% (*w*/*v*) Oil red O solution (60% isopropanol, 40% water) for 1 h at room temperature. The sections were washed with 60% isopropanol three times to remove unbound dye and photographed using a Nikon microscope.

### 2.12. Statistical Analysis

Data were expressed as the mean ± SD. The groups were compared using one-way ANOVA followed by Tukey’s post-hoc tests. *p* < 0.05 was considered statistically significant.

## 3. Results

### 3.1. EUL Prevents HFD-Induced Hepatic Steatosis

**Figure 1:**
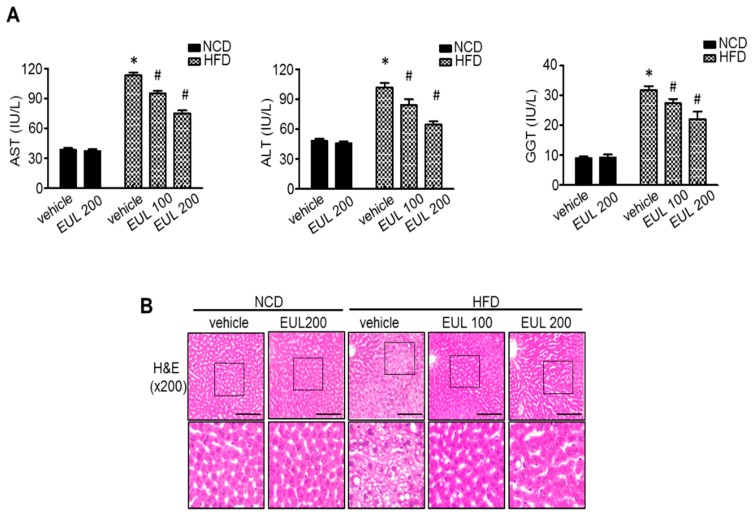
EUL ameliorates HFD-induced hepatic stress. Rats were fed a NCD or a HFD with or without administration of 100 mg/kg or 200 mg/kg EUL for 6 weeks, and sera and livers were harvested. (**A**) Biochemical analyses of serum samples. Serum levels of AST, ALT, and GGT in the different experimental groups. (**B**) Liver tissues retrieved 6 weeks after initial EUL administration and were subjected to H&E staining. Scale bars, 200 μm. Values are means ± SDs. (*n* = 6, *p < 0.05 versus NCD group, ^#^*p* < 0.05 versus HFD group). NCD, normal chow diet; HFD, high-fat diet; EUL, *Eucommia ulmoides* leaf extract; AST, aspartate aminotransferase; ALT, alanine aminotransferase. GGT, gamma-glutamyltransferase.

We examined the effect of EUL on hepatic lipotoxicity in rats fed with a HFD. Rats were fed a HFD to stimulate anabolism, for up to 10 weeks. This in vivo model is suitable for examining long-term signaling dynamics in a pathophysiologically relevant context. EUL was orally administered to rats at 100 or 200 mg EUL/kg/day. As shown in Figure 1A, compared with the normal diet group, serum AST, ALT, and GGT levels were significantly increased in the HFD-fed groups. Expression of the hepatic toxicity markers AST and ALT was downregulated by the extract (Figure 1A). To evaluate the effects of EUL on hepatic steatosis further, we stained the liver tissues with H&E to visualize lipid accumulation. As shown in Figure 1B, extensive macrovesicular steatosis surrounding the perisinusoidal areas and microvesicular steatosis were observed in HFD-fed rats. EUL treatment significantly decreased the accumulation of intracellular lipid droplets in HFD-fed rats.

**Figure 2:**
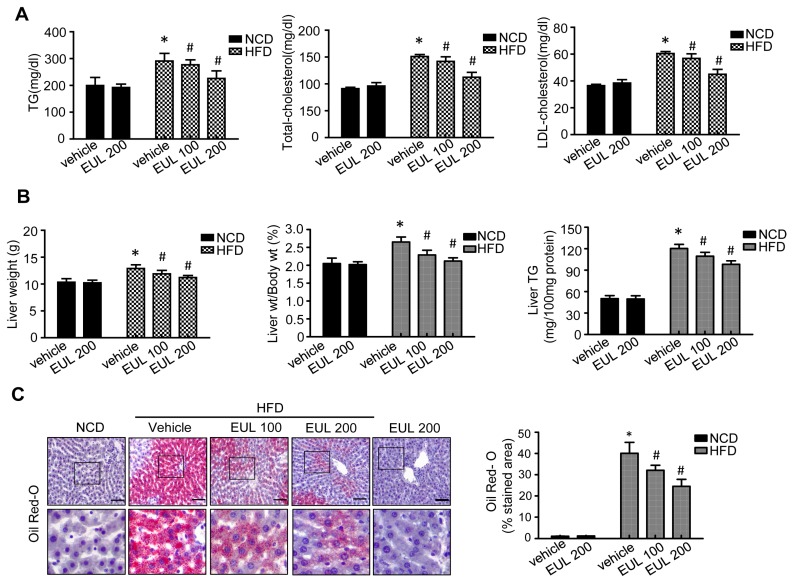
EUL prevents HFD-induced hepatic steatosis. Rats were fed a NCD or a HFD with or without administration of 100 mg/kg or 200 mg/kg EUL for 6 weeks, and sera and livers were harvested. (**A**). Biochemical analyses of serum samples. Serum levels of TG, TC, and LDL cholesterol in the experimental groups. (**B**) Liver weight, liver weight/body weight, and liver TG contents were measured 6 weeks after initial EUL administration (*n* = 6). (**C**) Liver tissues retrieved 6 weeks after initial EUL administration that were subjected to Oil red O staining. Scale bars, 200 μm. Values are means ± SDs. (*n* = 6, *p < 0.05 versus NCD group, ^#^*p* < 0.05 versus HFD group). NCD, normal chow diet; HFD, high-fat diet; EUL, *Eucommia ulmoides* leaf extract; TG, triglyceride; LDL, low-density lipoprotein.

As shown in Figure 2A, serum TG, TC, and LDL cholesterol levels showed significant increase in the HFD compared with the NCD group. After 6 weeks of treatment, serum TG, TC, and LDL levels showed significant reduction in the HFD plus EUL group compared with the HFD-fed rats. As shown in Figure 2B, significant differences in liver weight and liver weight/body weight gain were found between the NCD and the HFD group. Treatment with EUL significantly suppressed liver weight gain and the liver weight/body weight ratio in HFD-fed rats. Liver TG levels were significantly reduced in the HFD plus EUL compared to the HFD group. To evaluate the effects of EUL on hepatic steatosis and to visualize lipid accumulation, we stained the liver tissues with Oil Red O stain. As shown in Figure 2C, compared with the NCD group, lipid accumulation in the liver increased in HFD-fed rats, whereas treatment with EUL for 6weeks decreased the hepatic intracellular lipid accumulation in rats.

### 3.2. EUL Inhibits the mTORC1-ER Stress Response in HFD-fed Rats

**Figure 3:**
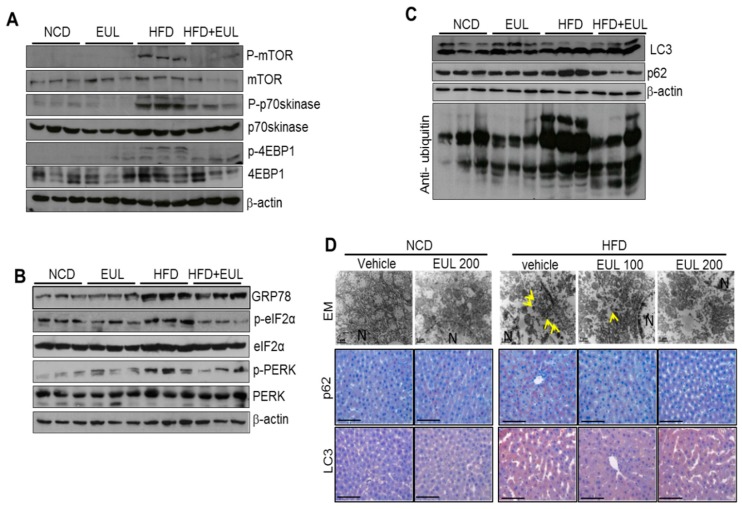
EUL inhibits ER stress in hyperlipidemic rats. (A) Experimental scheme. Rats were fed a HFD for 6 weeks, were given 100 mg/kg or 200 mg/kg EUL for 8 weeks, and were then sacrificed. Immunoblotting was performed using antibodies against p-mTOR, mTOR, p-p70S6K, p70S6K, p-4EBP-1, 4EBP-1, and β-actin. (**B**) Immunoblotting was performed using antibodies against GRP78, CHOP, p-PERK, PERK, p-eIF2α, eIF2α, and β-actin. (**C**) Immunoblotting was performed using antibodies against LC-3, p62, ubiquitin, and β-actin. (**D**) Representative EM images (upper panel) of anti-p62 immunostaining (middle panel), and anti-p62 immunostaining (lower panel). NCD, normal chow diet; HFD, high-fat diet; EUL, *Eucommia ulmoides* leaf extract; ER, endoplasmic reticulum.

The mTOR pathway mediates effects of obesity due to HFD in mammals [10,11]. To determine the mechanisms underlying HFD-induced hepatic lipid accumulation in vivo, we analyzed alterations in mTORC1 signaling, ER stress, and autophagy in the liver in EUL-treated and HFD-fed rats. HFD feeding resulted in the activation of mTORC1 signaling, as determined by increases in S6K and 4EBP-1 phosphorylation, which were attenuated by EUL treatment (Figure 3A). Next, we examined the significance of autophagy and ER stress in vivo. An excessive influx of free fatty acids leading to steatosis is regarded a potent inducer of ER stress and cell injury in obesity-induced NAFLD. Consistent herewith, livers from HFD-fed rats displayed a remarkable induction of eIF-2α and PERK phosphorylation, which triggered the ER stress response, and upregulation of downstream targets GRP78 and CHOP, all of which were attenuated by EUL treatment (Figure 3B). Next, we investigated whether the activation of ER stress signaling was accompanied by a change in autophagic flux signaling during the course of experimental NAFLD. Autophagy is one of the major downstream events regulated by mTORC1 [12]; autophagy activation can be suppressed by mTORC1. Electron microscopy revealed a reduction in double membrane structures (autophagosomes) and in the levels of p62 and LC 3-I to LC 3-II in EUL-treated compared with HFD-fed rats (Figure 3C,D).

### 3.3. EUL Suppresses Hepatic Oxidative Stress

**Figure 4:**
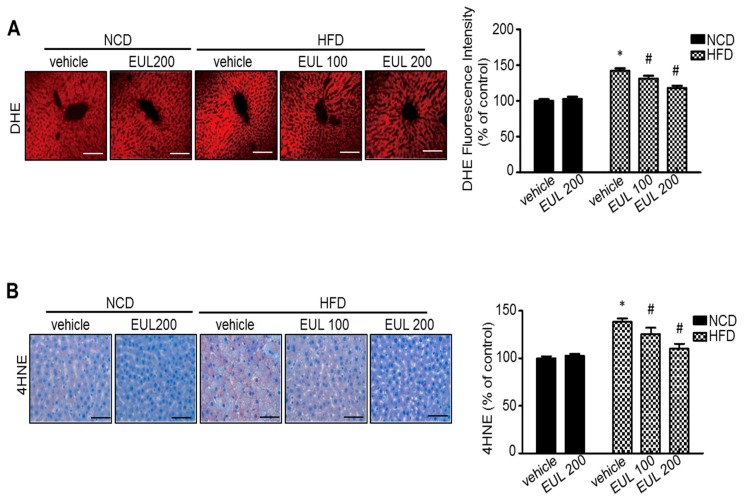
EUL inhibits oxidative stress in hyperlipidemic rats. Experimental scheme. Rats were fed a HFD for 6 weeks, were given 100 mg/kg or 200 mg/kg EUL for 8 weeks, and were then sacrificed. Liver DHE (**A**) and 4-HNE (**B**) levels. DHE is redox-sensitive fluorescent probe. 4-HNE are products of the lipid peroxidation cycle. Liver tissue was collected and was loaded with 5 μM dihydroethidium. Fluorescence images were acquired. Scale bars, 50 μm. Quantification of fluorescence intensity for superoxide levels in the liver. Values are means ± SDs. (*n* = 6, *p < 0.05 versus NCD group, ^#^*p* < 0.05 versus HFD group). NCD, normal chow diet; HFD, high-fat diet; EUL, *Eucommia ulmoides* leaf extract; and DHE, dihydroethidium.

**Figure 5:**
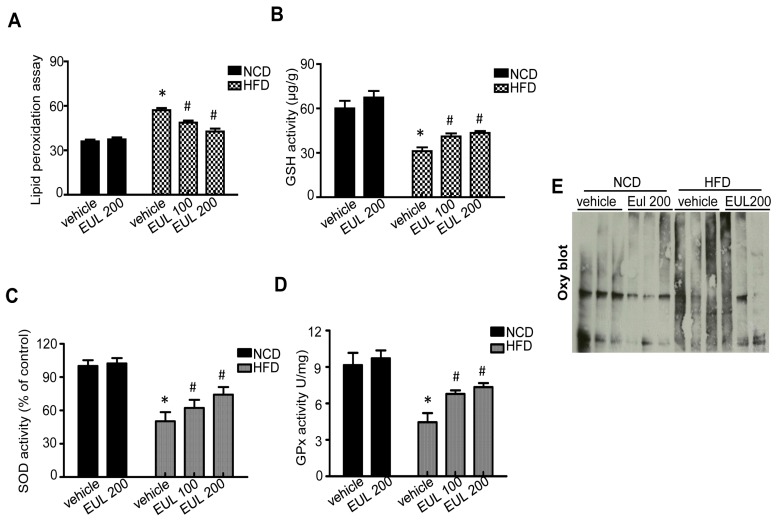
EUL regulates antioxidants in hyperlipidemic rats. Experimental scheme. Rats were fed a HFD for 6 weeks, were given 100 mg/kg or 200 mg/kg EUL for 8 weeks, and were then sacrificed. Liver MDA (**A**) and GSH (**B**) levels. SOD (**C**) and GPx (**D**) activity levels. (**E**) Oxidized protein in liver-tissue lysates was assessed by an OxyBlot assay. Values are means ± SDs. (*n* = 6, *p < 0.05 versus NCD group, ^#^*p* < 0.05 versus HFD group). NCD, normal chow diet; HFD, high-fat diet; EUL, *Eucommia ulmoides* leaf; MDA, malondialdehyde; SOD, superoxide dismutase; GPx, glutathione peroxidase; GSH, glutathione; Oxy; oxidized protein.

To determine whether EUL affects the antioxidative capacity, the effects of EUL treatment on several primary antioxidant defense components were measured. As shown in Figure 4A, in the HFD group, superoxide production was increased in the liver. When MDA was determined as a biomarker of lipid peroxidation, significant declines in hepatic MDA were observed in the EUL groups. Regional hepatic oxidative stress was assessed by DHE staining of superoxide production in the liver. Treatment with EUL suppressed the HFD-induced increase in DHE-stained cells (Figure 4A). Next, we assessed the antioxidative action of 4-hydroxynonenal (4-HNE), which had enhanced enzyme activity in the presence of EUL (Figure 4B). Increased levels of ROS induced membrane lipid peroxidation and the production of associated byproducts, such as MDA and 4-HNE [13]. However, in the HFD plus EUL group, MDA was controlled at a level comparable to that in the normal control group (Figure 5A). The GSH levels were elevated substantially with EUL, and the highest level was detected in the 200 mg EUL group (Figure 5B). SOD and GPx activities were significantly higher in the EUL than in the HFD groups (Figure 5C,D). The protein carbonyl group is a biomarker of oxidative stress [14]. To test whether the effects of EUL on oxidative damage were mediated by protein carbonyl, we used 4%–12% Bis-Tris gel electrophoresis to resolve proteins and then conducted western blot analysis using an anti-dinitrophenyl antibody. Liver-protein-carbonyl expression levels in HFD rats were strongly increased compared to those in the NCD group. However, EUL-treated rats had significantly lower protein-carbonyl expression levels in the liver than HFD rats (Figure 5E).

### 3.4. EUL Enhances Lysosomal Enzyme Activation

**Figure 6:**
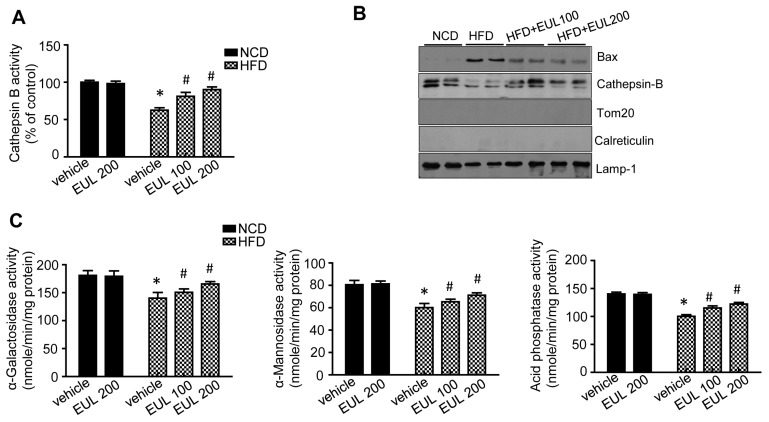
EUL regulates HFD-induced lysosomal Bax localization and lysosomal enzyme activation. (**A**) Cathepsin B activity in liver lysates. (**B**) Western blotting with antibodies against Bax, cathepsin B, LAMP-1, and tubulin. (**C**) Liver levels of α-galactosidase, α-mannosidase, and acid phosphatase. Values are means ± SDs. (*n* = 6, *p < 0.05 versus NCD group, ^#^*p* < 0.05 versus HFD group). NCD, normal chow diet; HFD, high-fat diet; and EUL, *Eucommia ulmoides* leaf extract.

The capability of EUL to enhance lysosomal function in HepG2 cells has been established [15]. Because localization of Bax in the lysosomes induces lysosome permeabilization and subsequently, leakage of the acidic protease cathepsin, which leads to cell death, we investigated the expression of cathepsin B. Cathepsin B activity in the lysosomes was inhibited by treatment with each component (Figure 6A). To confirm the regulation of lysosomal localization of Bax and the subsequent release of cathepsin B in HFD and/or EUL-supplemented rats, lysosomal and cytosolic fractions were analyzed. Translocation of Bax to the lysosomes and leakage of cathepsin B from the lysosomes induced by HFD were clearly reversed by supplementation with EUL (Figure 6B). Finally, the activities of the lysosomal enzymes α-galactosidase, α-mannosidase, and acid phosphatase in HFD-fed rats were rescued by cotreatment with EUL (Figure 6C).

### 3.5. EUL Prevents Palmitate-Induced Lipid Accumulation by Inhibiting the mTORC1-ER Stress Pathway in HepG2 Cells

**Figure 7:**
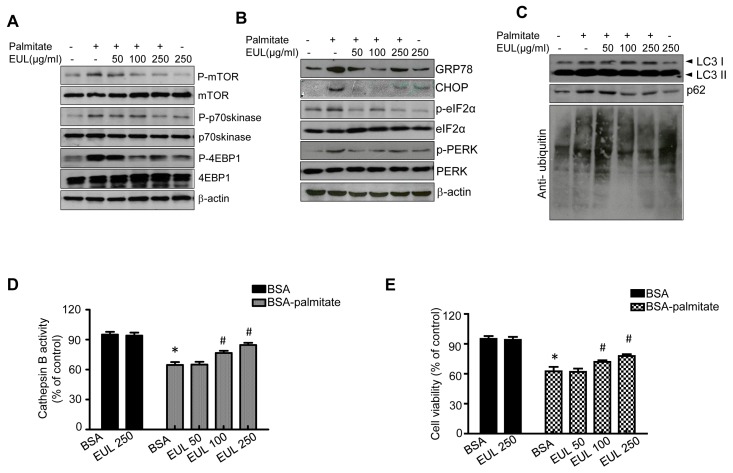
Effects of EUL on ER stress and lysosomal function in HepG2 cells. HepG2 cells were treated with 50 μg/mL, 100 μg/mL, or 250 μg/mL EUL for the indicated periods, and the expression of proteins of interest was determined by western blotting. (**A**) Immunoblotting using antibodies against p-mTOR, mTOR, p-p70S6K, p70S6K, p-4EBP-1, 4EBP-1, and β-actin. (**B**) Immunoblotting using antibodies against GRP78, CHOP, p-PERK, PERK, p-eIF2α, eIF2α, and β-actin. (**C**) Immunoblotting using antibodies against LC-3, p62, ubiquitin, and β-actin. (**D**) HepG2 cells were incubated with the indicated concentrations of EUL for 24 h, and cathepsin B activity was determined. (**E**) HepG2 cells were treated with 50 μg/mL, 100 μg/mL, or 250 μg/mL EUL for the indicated periods, and cytotoxicity was determined using the MTT assay. Values are means ± SDs. *n* = 3, *p < 0.05 versus control. ^#^*p* < 0.05 versus palmitate. EUL, *Eucommia ulmoides* leaf extract, BSA; bovine serum albumin.

We next investigated the potential of EUL to overcome the blockade of autophagic flux by fatty acid overload by conducting in vitro experiments using HepG2 cells treated with palmitate, which inhibited the lysosomal cell death pathway [15]. To determine whether enhanced autophagosome accumulation, mTORC activation, and ER stress contributed to palmitate-induced lipid accumulation and oxidative stress, we measured the expression of critical proteins involved in mTOR signaling, ER stress, and autophagy by western blotting. As shown in Figure 7A, palmitate treatment induced significant increases in p-mTOR, p-p70skinase, and p-4EBP-1. In contrast, treatment with EUL significantly reduced the phosphorylation of these proteins in HFD rats. Regarding ER stress, palmitate treatment induced GRP78, PERK, CHOP, and eIF2a in HepG2 cells. Cotreatment with EUL reduced these effects of palmitate; it significantly blocked PERK and eIF2α phosphorylation and suppressed expression of GRP78 and CHOP (Figure 7B). Next, we investigated the effects of EUL treatment on autophagic activity in HepG2 cells. EUL treatment promoted the conversion of LC3-I to LC3-II in a dose-dependent manner. Further, EUL treatment increased the degradation of SQSTM1/p62 protein in a dose-dependent manner, as shown by a reduction in the SQSTM1/p62 turnover index, and autophagic flux was blocked as revealed by a significant decrease in the levels of polyubiquitinated proteins (Figure 7C). Cathepsin B activity in the lysosomes was inhibited by treatment with each component (Figure 7D). The effects of EUL on lipotoxicity during fatty acid accumulation were confirmed; EUL showed a dose-dependent protective effect against palmitate-induced cell death (Figure 7E).

### 3.6. The Major Component of EUL, Aucubin, and Another Well-Known Major Component of EUO Cortex, Geniposide Regulate Lysosomal Function in HepG2 Cells

**Figure 8:**
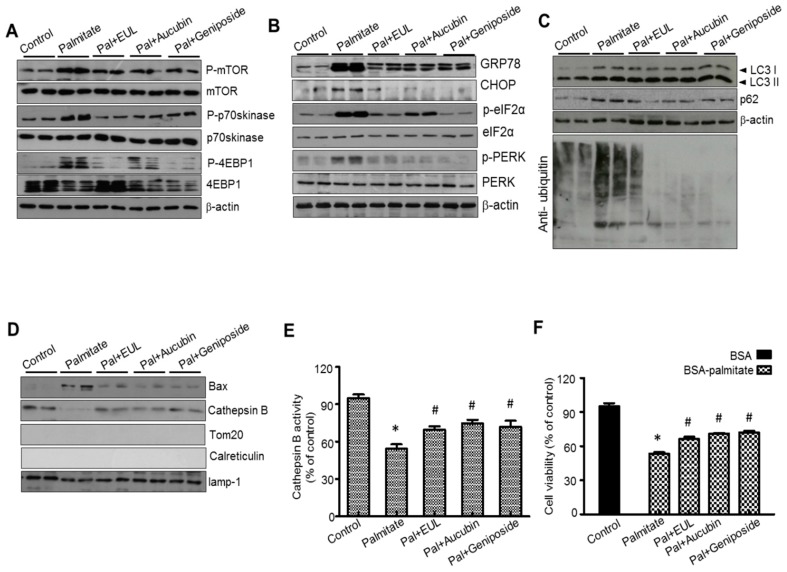
The major component of EUL, aucubin and one of the main components of EUO cortex, geniposide, regulate palmitate-induced steatosis in HepG2 cells. (**A**) HepG2 cells were treated with 250 μg/mL EUL, 25 μg/mL aucubin, or 25 μg/mL geniposide for 24 h, and the expression of proteins of interest was determined by western blotting. Immunoblotting using antibodies against p-mTOR, mTOR, p-p70S6K, p70S6K, p-4EBP-1, 4EBP-1, and β-actin. (**B**) Immunoblotting using antibodies against anti-GRP78, CHOP, p-PERK, PERK, p-eIF2α, eIF2α, and β-actin. (**C**) Immunoblotting using antibodies against LC-3, p62, ubiquitin, and β-actin. (**D**) HepG2 cells were incubated with 250 μg/mL EUL, 25 μg/mL aucubin, or 25 μg/mL geniposide for 24 h, and cathepsin B activity was determined. (**E**) HepG2 cells were treated with 250 μg/mL EUL, 25 μg/mL aucubin, or 25 μg/mL geniposide for 24 h, and (**F**) cytotoxicity was determined using the MTT assay. Values are means ± SD. *n* = 3, *p < 0.05 versus control. ^#^*p* < 0.05 versus palmitate. EUL, *Eucommia ulmoides* leaf extract and EUO cortex, *Eucommia ulmoides* cortex extract; BSA, bovine serum albumin.

In our previous report [8], aucubin was established as a standard component as well as a major effective component, whereas in other studies, geniposide had been also considered as a main component to explain the biological function of EUO especially EUO cortex [16]. Therefore, we tested the effects of aucubin and geniposide on palmitate-induced lipotoxicity in HepG2 cells. First, we examined mTORC activation in the presence of EUL, aucubin, and geniposide. Palmitate significantly reduced the phosphorylation of mTOR, p-p70s6kinase, and 4EBP-1, whereas EUL, aucubin, and geniposide reduced mTORC activation in HepG2 cells (Figure 8A). Cotreatment with aucubin or geniposide reduced palmitate-induced events; it significantly blocked PERK and eIF2α phosphorylation and decreased the expression of GRP78 and CHOP (Figure 8B). When we investigated the effects of EUL, aucubin, and geniposide on autophagic activity of palmitate-treated HepG2 cells, we found that EUL, aucubin, and geniposide promoted the conversion of LC3-I to LC3-II and SQSTM1/p62 protein degradation, and decreased the levels of polyubiquitinated proteins (Figure 8C). To confirm the regulation of lysosomal localization of Bax and the subsequent release of cathepsin B in aucubin- or geniposide e-treated cells, lysosomal and cytosolic fractions were analyzed. Translocation of Bax to the lysosomes and cathepsin B leakage were recovered by treatment with aucubin or geniposide (Figure 8D). Cathepsin B activity in the lysosomes was inhibited by treatment with each component (Figure 8E). Aucubin and geniposide showed a protective effect against palmitate-induced cell death (Figure 8F).

## 4. Discussion

In this study, we examined the effects of EUL supplementation on HFD-induced NAFLD and hepatic steatosis. EUL-associated lysosomal activation is a main mechanism underlying the protective effect of EUL against NAFLD, in which mTORC1 activation and the subsequent protein- folding load, i.e., ER stress, are involved. Isoflavonoid-rich EUL also controls the hypernutritional stress-associated ROS and ER stress-linked redox imbalance and ROS production, ultimately regulating ER stress and hepatic dyslipidemia. The in vitro and in vivo experiments showing the effect of EUL on lipid-induced dysmetabolism also revealed that EUL also regulated mTORC1 status, thus controlling ER stress and autophagy. mTORC1 activation and protein folding represent the main pathological mechanisms of high-nutrient-associated hepatic metabolism. It is known that a high level of free fatty acids leads to constitutive activation of mTOR signaling, a process related to the development of diseases such as diabetes and obesity [17,18]. Along with the inhibitory effect against mTOR, EUL supplementation suppressed ER stress in NAFLD pathogenesis. ER stress is one of the major features of pathological conditions associated with obesity and NAFLD [19,20,21]. Furthermore, we found an accumulation of polyubiquitinated proteins and p62 and an increase in the LC3-II/LC3-1 ratio in hepatocytes of HFD-fed rats, which probably led to chronic UPR activation, as demonstrated by elevated PERK and eIF2a phosphorylation and increased GRP78 and CHOP protein expression, reflecting ER overload. Whereas polyubiquitinated proteins and p62 levels increased in HFD-fed rats, indicating the defective autophagic function of EUL abolished the lipid accumulation and ER stress response induced by excess nutrients, suggesting that the PERK/eIF2α UPR branch, a critical regulator of lipid metabolism, is controlled via EUL-associated autophagy regulation. Autophagy maintains ER function by removing dysfunctional lysosomes. Based on the finding that autophagy was suppressed by a HFD and EUL enhanced lysosome function, we concluded that EUL reduced ER stress by increasing lysosome function under hyperlipidemia. In previous studies, we explored the effects of EUL on palmitate-treated hepatocytes and NAFLD in rats. It has been reported that EUO cortex extract decreased serum AST, ALT, LDH, and ALP levels in a liver damage model [15,22]. Consistently, as a protective mechanism against hepatic cell death, EUL regulates the localization of fatty acid-related lysosomal Bax and the basic mechanism of cathepsin B leakage and cell death in the NAFLD model (Figure 7E). Another important finding of the present study was that EUL supplementation alleviated hepatic steatosis by controlling hepatic dysmetabolism-associated ROS accumulation, which was also linked to ER redox disturbance and ER stress. EUL contains various bioactive substances, including lignans, phenols, triterpenes, organic acids, and various antioxidants [23], and has been used in foods and pharmaceuticals because of its pharmacological effects. We previously assessed whether EUL prevented fatty liver disease caused by HFD in vivo through inhibiting ER stress and reducing ROS production [15]. HFD provided an ideal model for studying the effect of EUL on lipid metabolism due to NAFLD, as oxidative stress resulted in the accumulation of lipid peroxides and the loss of antioxidant enzyme capacity [24,25]. Our results showed that HFD elevated oxidative stress and ER stress in the liver, as indicated by decreases in the hepatic 4-HNE level and GSH/GSSG ratio and in serum MDA, and the induction of the UPR, i.e., PERK, CHOP, and GRP78 expression. EUL prevented HFD-induced oxidative stress by upregulating the activity of antioxidant enzymes, including GSH, SOD, and GPx (Figure 5B–D). In addition, EUL was also found to control ER stress alleviating ER-oxido-reduction folding disturbance, and subsequently diminishing ER stress-associated ROS production. These findings implicate that EUL ameliorates hepatic steatosis through mitigating oxidative stress, whether or not ER stress is involved, suggesting that EUL might be a potential inhibitor of lipid metabolic imbalance. Further, EUL prevents HFD-induced dyslipidemia in rats. In hamsters, HFD-induced hyperlipidemia was prevented by treatment with EUL at 0.175 g/100 g diet [26]. Diet-induced obesity in Sprague-Dawley rats was prevented by dietary supplementation of 0.25, 0.5, or 1 g/kg EUO cortex extract in our recent studies [15,22]. Based on these previous reports, we applied the same dose of EUL to treat the dyslipidemic condition. In HFD-fed rats, hepatocyte dysfunction was accompanied by changes in lipid metabolism, which involved anabolic signaling, mTOR. Considering that aucubin has potential role in diverse biological functions and that it is a main component of EUL, it was considered as the standard component for the stablility and quality of EUL as well as the main effective component [8]. In addition to aucubin, geniposide has been frequently studied as an effective component of EU, especially the EUO cortex. Therefore, we examined the consistent role of the two effective components in this study (Figure 8). A recent study showed that EUL and its compounds have various biochemical activities, such as anti-hyperlipidemic, anti-atherosclerosis, antioxidant, anti-diabetic, and immunomodulatory effects [17]. The present study revealed that EUL is rich in lignans, iridoids, flavonoids, and polysaccharides [27,28]. In the present study, we used crude EUL, but recently, quality control data of the pure components, especially aucubin, have been reported [29]. Further studies are required to investigate the biological effects of aucubin and the other polyphenols using the NAFLD model. Our previous study suggested that aucubin and geniposide suppressed cell death by regulating lysosomal Bax translocation and lysosome function [15]. Along with EUL, mTOR signaling in hepatocytes was inhibited in the presence of geniposide or aucubin (Figure 8A). Consistently, geniposide and aucubin inhibited palmitate-induced autophagy flux inhibition (Figure 8C). Furthermore, our in vitro data indicated that the major components reduced the expression of the ER stress-related proteins PERK, eIF2α, GRP78, and CHOP, and lysosomal Bax translocation, recovering the cellular endogenous cathepsin B activity and cell viability. Thus, aucubin and geniposide, also have similar effects on the regulation of mTOR, autophagy, related unfolded-protein loading, ER stress, and cell death.

## 5. Conclusions

In an HFD-induced NAFLD model, EUL exerts positive effects on hepatocyte damage caused by HFD by reducing oxidative stress and restoring lipid metabolism, leading to lysosomal activation and the alleviation of ER stress. The results of this study may lead to the development of novel strategies to prevent complications of NAFLD.
